# A Bayesian bivariate spatial modeling framework for stage-specific cancer incidence and targeting of screening efforts

**DOI:** 10.1093/aje/kwag106

**Published:** 2026-05-19

**Authors:** Qinyun Lin, Carl Bonander, Ulf Strömberg

**Affiliations:** School of Public Health and Community Medicine, Institute of Medicine, Sahlgrenska Academy, University of Gothenburg, Gothenburg, Sweden; School of Public Health and Community Medicine, Institute of Medicine, Sahlgrenska Academy, University of Gothenburg, Gothenburg, Sweden; Karlstad Business School, Karlstad University, Karlstad, Sweden; School of Public Health and Community Medicine, Institute of Medicine, Sahlgrenska Academy, University of Gothenburg, Gothenburg, Sweden

**Keywords:** cancer screening, disease mapping, BYM2, INLA, stage-specific cancer, small area estimation, Bayesian bivariate spatial model, diagnostic inequality

## Abstract

Geographic differences in cancer stage at diagnosis may reflect both variation in underlying cancer risk and variation in the extent of early detection through screening or other diagnostic testing. Mapping both late-stage incidence (as an indicator of disease burden) and late-minus-early difference (as an indicator of insufficient diagnostic activity relative to the underlying disease burden) provides an empirically grounded way to identify small areas (neighborhoods) where enhanced early detection may yield the greatest benefits. We introduce a Bayesian bivariate spatial modeling framework for jointly analyzing early- and late-stage cancer incidence, allowing for both spatial correlation across areas and outcome correlation within areas to improve small-area estimation. Using population-based registry data aggregated at the small-area level, we estimate age-standardized incidence rates of early- and late-stage cancers across the study area and propose an inferential approach to identify priority sub-areas. We compare single-outcome and multivariate spatial formulations via Integrated Nested Laplace Approximation. We demonstrate the approach using Swedish register data on prostate cancer linked to population data at the small area level. The framework can be extended to other diseases with correlated outcomes or multiple severity levels, as well as to other settings.

## Introduction

Early detection remains central to reducing cancer mortality, yet substantial geographic and socioeconomic disparities persist in when and how cancers are diagnosed worldwide. Differences in healthcare organization, diagnostic activity, and access to screening programs contribute to striking variation in cancer stage at diagnosis across regions and population groups.^1-5^ Studies have shown that people living in socioeconomically deprived or rural areas are more likely to be diagnosed at advanced stages, reflecting unequal access to timely diagnostic services.^3^^,^^4^^,^^6^

These disparities often operate at a fine geographic scale. Small-area (neighborhood) analyses capture variation in socioeconomic conditions, healthcare accessibility, and urbanization that national or regional statistics may obscure. Identifying where diagnostic activity is insufficient relative to local disease burden can guide targeted interventions to improve early detection. However, most previous spatial studies have modeled either early-stage or late-stage cancer incidence in isolation,^6-8^ limiting our understanding of how shared contextual determinants shape both outcomes simultaneously.

Late-stage cancer incidence reflects the public health burden of advanced disease, whereas early-stage incidence, which often includes asymptomatic cases detected through screening or other diagnostic encounters, may serve as a proxy for diagnostic activity when interpreted alongside late-stage incidence.^4^^,^^9^ Together, these measures provide complementary perspectives on population-level cancer detection and its inequities. By jointly examining their spatial distribution, one can identify areas that experience both a high burden of advanced disease and a low yield of early detection, suggesting potential gaps in diagnostic activity.

Bayesian hierarchical modeling provides an appealing solution for small-area estimation, as it enables borrowing of information across neighboring areas and correlated outcomes, stabilizing estimates where case counts are sparse.^10-12^ Extending this framework to a bivariate spatial framework allows simultaneous modeling of early- and late-stage incidence, capturing both spatial dependence and cross-outcome correlation in a unified structure.

A related Bayesian framework models stage at diagnosis as an ordinal outcome using cumulative or sequential formulations,^13^ focusing on modeling the distribution of stages conditional on total incidence and estimating stage-specific probabilities or relative risks. While highly valuable for understanding stage patterns, this formulation does not, by itself, impose a distinction between late-stage disease burden and early-stage detection activity, as this depends on how model outputs are subsequently conceptualized. Our approach is motivated by a conceptual framework in which late-stage incidence reflects disease burden and early-stage incidence serves as a proxy for diagnostic activity, enabling joint evaluation of burden and potential diagnostic insufficiency at the population level.

Previous multivariate disease-mapping frameworks have primarily focused on jointly modeling related health outcomes to improve precision and identify shared spatial risk structure.^14-17^ Building on the shared-component formulation by Held and Best^14^ and subsequent extensions, including applications to cancer incidence and mortality^18^ and recent developments incorporating flexible shared spatiotemporal components,^19^ these Bayesian models combine shared and outcome-specific spatial or spatiotemporal fields to capture both common and disease-specific geographic variation. Extending this Bayesian multivariate tradition, our study develops and illustrates a bivariate spatial modeling framework to stage-specific incidence of a single cancer type.

## Conceptual framework

The conceptual framework is organized around the mechanism through which screening affects cancer outcomes. Our focus is on screening aimed at early cancer detection, as for breast and prostate cancer where screening mainly shifts the stage at diagnosis rather than reducing underlying cancer incidence.^20^^,^^21^ For some cancers, including cervical and colorectal cancer, preventive effects of screening should also be considered.^22-24^ In such settings, an alternative conceptual distinction can be made by treating total incidence, rather than late-stage incidence, as the relevant measure of disease burden.

We build on earlier work to argue that understanding cancer detection requires distinguishing between the burden of disease and the level of diagnostic activity within a population.^4^^,^^9^ Geographic differences in stage at diagnosis across small areas may reflect both variation in (1) the underlying risk of developing cancer and (2) the extent to which early detection occurs through organized screening or other diagnostic testing. Late-stage cancer incidence can be viewed as an indicator of the public health burden of advanced disease, whereas early-stage incidence, often including asymptomatic cases detected through organized screening or opportunistic testing, serves as a proxy for diagnostic activity.^4^^,^^9^ Considering these 2 measures jointly provides a more informative perspective on population-level cancer detection and helps identify geographic areas where early detection may be insufficient relative to disease burden.

We use the terms late-stage and early-stage to denote clinically meaningful categories of tumor progression at diagnosis. For prostate cancer, late stage may correspond to locally advanced or metastatic cancer at diagnosis and early stage to low-risk cancer.^9^ Although these definitions vary across cancer types, the late/early distinction is broadly applicable when targeting screening efforts aimed at early cancer detection.

Residual spatial variation in stage-specific incidence may reflect a combination of contextual and individual-level influences. Some contextual factors, such as environmental or behavioral risk exposures, may increase overall incidence and thereby elevate both early- and late-stage rates within the same areas.^25-27^ Other contextual factors, particularly those related to healthcare access, diagnostic capacity, and screening activity, can have the opposite effect by determining when cancers are detected, reducing early diagnosis where late-stage burden is high.^28^^,^^29^ Joint consideration of early- and late-stage incidence can therefore help identify populations and areas where diagnostic activity may be insufficient. At the individual level, risk and access factors may also interact. For example, racial groups with higher intrinsic cancer risk may live in neighborhoods with limited diagnostic resources, reinforcing these spatial disparities.^30^ These processes can manifest as both spatially structured (geographically correlated) and unstructured (local or random) components, motivating a bivariate spatial model that partitions residual variation into correlated structured and unstructured effects.

**Figure 1:**
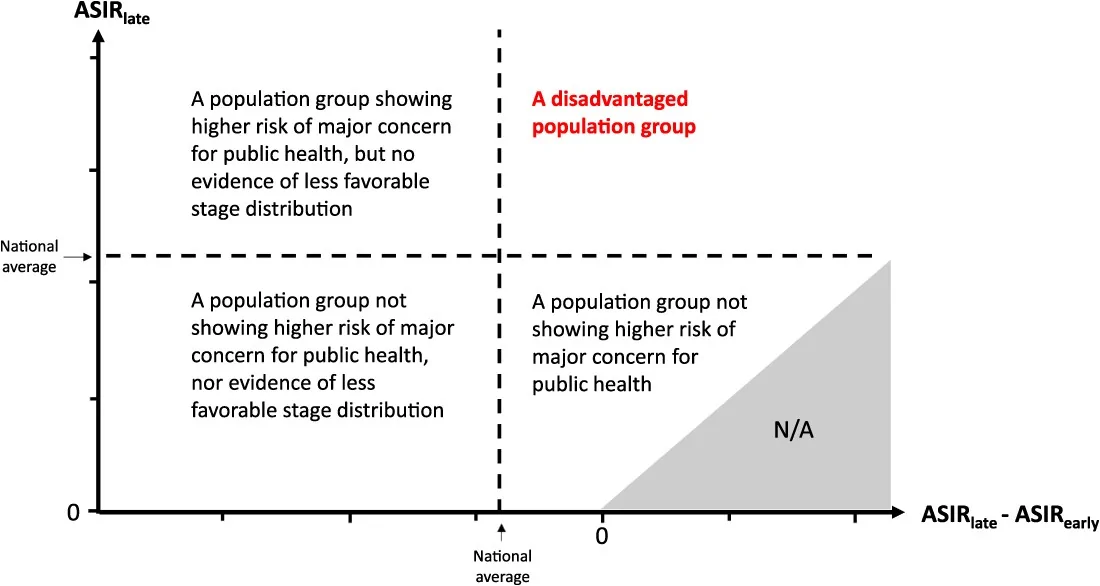
Conceptual framework linking age-standardized incidence rates of late- and early-stage—a basis for targeting population groups for screening efforts aimed at early cancer detection. Areas or population groups positioned in the upper-right quadrant exhibit both elevated late-stage incidence and a larger difference between late- and early-stage rates, indicating a combination of high disease burden and insufficient early detection. Here, we labeled such as disadvantaged. Groups in the upper-left quadrant show high burden without evidence of diagnostic disadvantage, while those in the lower quadrants are of less immediate public-health concern. There are 2 conditions: ASIR_late_ > 0 and ASIR_late_—ASIR_early_ < ASIR_late_ (the gray triangle indicates values not applicable). Abbreviation: ASIRs, age-standardized incidence rates.

Let ${\mathrm{ASIR}}_{\mathrm{late},i}$ and ${\mathrm{ASIR}}_{\mathrm{early},i}$ represent the model-based age-standardized incidence rates of late- and early-stage cancer, respectively, for small area *i=1,2,…, n*. Areas with both higher ${\mathrm{ASIR}}_{\mathrm{late},i}$ and larger differences ${\mathrm{ASIR}}_{\mathrm{late},i}-{\mathrm{ASIR}}_{\mathrm{early},i}$ relative to national averages indicate a combination of elevated disease burden and limited diagnostic activity. This relationship can be represented within a 2-dimensional framework that locates population groups or areas according to their estimated ${ASIR}_{late,i}$ (vertical axis) and ${\mathrm{ASIR}}_{\mathrm{late},i}-{\mathrm{ASIR}}_{\mathrm{early},i}$ (horizontal axis) (Figure 1). This conceptual framework provides a population-based rationale for identifying priority areas for early detection interventions. Statistically, it motivates a joint modeling approach that treats early- and late-stage incidence as correlated outcomes reflecting related but distinct processes. By jointly modeling both outcomes, information can be shared across stages to improve small-area estimation and to characterize spatial patterns in diagnostic activity relative to disease burden.

## Methodological framework

We now outline the modeling framework, beginning with single-outcome spatial models and then extending to a bivariate formulation that incorporates flexible fixed effects and supports inference on areas with potentially insufficient diagnostic activity relative to disease burden. Our overall model hierarchy (Models 1-4) builds on established Bayesian multivariate and shared-component disease-mapping frameworks and extends them within the Besag–York–Mollié 2 (BYM2) formulation.^12^^,^^31^ This extension distinguishes spatially structured and unstructured components for each outcome, providing a unified inferential framework for quantifying geographic heterogeneity and identifying spatial disparities between early- and late-stage incidence.

### Single-outcome spatial models

We first consider separate spatial models for early- and late-stage cancer incidence. Let *i=1,2,…, n* index small areas, *j=1,2,…, J* index strata (eg, combinations of age group and calendar year, as applicable), and *k=1,2* denote the 2 outcomes, with *k=1* representing early-stage and *k=2* representing late-stage cancer incidence. For each outcome *k*, the observed number of cases ${y}_{ijk}$ in area *i* stratum *j* follows a Poisson model: ${y}_{ijk}\sim \mathrm{Poisson}\left({E}_{ijk}{\lambda}_{ijk}\right)$, $\log \left({\lambda}_{ijk}\kern0em \right)={\alpha}_k+{X_{ijk}}^T\kern0em {\beta}_k+{\gamma}_{ik}$, where ${E}_{ijk}$ denotes the population at risk and serves as the offset term, while ${\lambda}_{ijk}$ represents the underlying incidence rate for outcome *k* in stratum *j* and area *i*. Our modeling framework also applies to the other commonly used disease-mapping formulation in which ${E}_{ik}$ represents the expected case numbers under reference rates for each small area and ${\lambda}_{ik}$ is interpreted as a relative risk. The latent spatial component ${\gamma}_{ik}$ follows the BYM2 specification^12^^,^^31^: ${\gamma}_{ik}=\sqrt{1-{\phi}_k}{v}_{ik}+\sqrt{\phi_k}{u}_{ik}$, where ${v}_{ik}$ captures unstructured heterogeneity and ${u}_{ik}$ is a structured spatial effect following an intrinsic conditional autoregressive (ICAR) model, implying that neighboring areas tend to have similar risks. The parameter ${\phi}_k\in \left[0,1\right]$ governs the proportion of spatial variance attributed to the structured ICAR component versus the unstructured noise. Each stage *k* is modeled independently (Model 1), providing spatially smoothed, stage-specific incidence rates and serving as the baseline for evaluating the gains from joint bivariate modeling.

### Specification of fixed effects

The fixed-effect components ${X_{ijk}}^T\kern0em {\beta}_k$ captures the influence of covariates such as age, sex, and calendar year on the stage-specific incidence rates. In the single-outcome model (Model 1), all covariates are estimated separately for early- and late-stage outcomes, allowing complete flexibility in their effects.

In the bivariate framework, selected covariates can be specified with outcome-specific effects through interaction terms (eg, age-by-outcome), allowing the 2 outcomes to follow distinct covariate gradients while maintaining a coherent joint structure. Covariates without strong evidence of differential effects may be modeled as common across outcomes to improve model parsimony.

### Bivariate spatial models

To capture potential correlation between early- and late-stage incidence across neighborhoods, we extended the single-outcome models described above into a bivariate spatial framework. The stage-specific Poisson models share the same structure as above, but their spatial random effects ${\gamma}_{i1}$ and ${\gamma}_{i2}$ are modeled jointly to allow borrowing of information across outcomes. We present 3 alternative specifications (Models 2-4), each representing a distinct epidemiologic assumption about how the 2 outcomes are spatially related.

#### Model 2: Shared structured spatial surface model

Both outcomes share a single structured spatial component ${u}_i$ that captures spatially correlated variation common to early- and late-stage incidence, while retaining outcome-specific unstructured IID noise term to represent residual heterogeneity. Formally, $\log \left({\lambda}_{ik}\kern0em \right)={\alpha}_k+{X_{ik}}^T{\beta}_k+{u}_i+{v}_{ik}$, where ${u}_i$ follows an ICAR prior common to both outcomes and ${v}_{ik}$ are independent across small areas and outcomes. This formulation corresponds to a shared structured field but outcome-specific unstructured variation. It is based on the hypothesis that a single spatial process (eg, region-level environmental or healthcare system characteristics) drives large-scale geographic patterns in both outcomes, whereas local random factors differ by outcome (eg, diagnostic practices and administrative variation).

#### Model 3: Exchangeable correlated spatial fields

The random effects for early and late stages are assumed to follow a bivariate normal distribution with a common correlation parameter *ρ*. Both the structured (${u}_{ik}$) and unstructured (${v}_{ik}$) components share this same *ρ*, implying a single degree of cross-outcome association: $\mathrm{Cov}\left({\gamma}_{i1},{\gamma}_{i2}\kern0em \right)=\rho \sqrt{1-\phi }{\Sigma}_v+\rho \phi{\Sigma}_u$, where *ϕ* is the proportion of structured variance in the BYM2 model. Thus, Model 3 assumes 2 distinct spatial surfaces that are similarly shaped across the map, differing in magnitude but linked by a constant correlation. Epidemiologically, it represents partially shared spatial determinants such as overlapping environmental and healthcare influences that are not identical for both outcomes. This specification parallels the borrowing-strength framework of Riebler et al.^32^ linking related latent effects via a shared correlation parameter.

#### Model 4: Separate structured and unstructured components

Model 4 provides the most flexible specification, allowing distinct structured (${u}_{ik}$) and unstructured (${v}_{ik}$) components for each outcome, each with its own cross-outcome correlation parameter. This formulation enables the data to determine whether correlation arises primarily from spatially structured processes (eg, shared environmental exposures for which neighboring areas tend to have similar risks) or from heterogeneous local variation (eg, diagnostic or administrative factors). Formally, we have: $\mathrm{cor}\left({u}_{i1},{u}_{i2}\kern0em \right)={\rho}_u$ and $\mathrm{cor}\left({v}_{i1},{v}_{i2}\kern0em \right)={\rho}_v$. Models 2 and 3 are nested within Model 4 as special cases: if the 2 structured spatial components are constrained to be identical (${u}_{i1}={u}_{i2}$) while the unstructured components remain independent (${v}_{i1}\perp{v}_{i2}$), Model 4 reduces to Model 2. If both the structured and unstructured components are constrained to have a common cross-outcome correlation (${\rho}_u={\rho}_v=\rho$), Model 4 reduces to Model 3. By estimating both correlation parameters, Model 4 distinguishes between spatially structured and heterogeneous, unstructured sources of cross-outcome association, offering a comprehensive and interpretable bivariate extension of the BYM2 model.

Model estimation can be performed using Integrated Nested Laplace approximation (INLA)^11^^,^^33^ within the BYM2 framework.^12^^,^^31^ In practice, Model 4 is implemented using 2 correlated Besag (structured) and 2 correlated IID (unstructured) fields in INLA, consistent with the BYM2 decomposition of spatial effects into structured (ICAR) and unstructured components (see Table S1 for details). Penalized-complexity (PC) priors are applied to the precision parameters (τ) of all random effect components to favor more parsimonious specifications unless the data supported additional complexity.^34^ Model performance among the bivariate specifications (Models 2-4) can be compared using the Deviance Information Criterion (DIC),^35^ a standard Bayesian measures of predictive accuracy and model complexity implemented in INLA.

### Inferential procedures for identifying disadvantaged areas

Following model estimation, posterior estimates from the bivariate spatial model can be used to identify areas where diagnostic activity may be insufficient relative to disease burden. For each stratum, posterior estimates of age-specific incidence rates for early- and late-stage outcomes are obtained from the fitted model. ASIRs are then calculated by directly weighting the model-predicted age-specific incidence rates (posterior medians) according to the national age distribution (direct internal standardization). This step yields absolute, age-adjusted stage-specific incidence rates and their difference as comparable population-level summary measures for identifying priority areas. The posterior probability that each area exceeds the national average for both ${\mathrm{ASIR}}_{\mathrm{late}}$ and ${\mathrm{ASIR}}_{\mathrm{late}}-{\mathrm{ASIR}}_{\mathrm{early}}$ can then be computed (cf. Figure 1). Considering previous recommendations,^10^^,^^36^ a threshold of 0.8 is used to identify priority areas for interventions aimed at increasing diagnostic activity. This inferential framework provides a general approach for jointly evaluating disease burden and diagnostic activity while accounting for spatial dependence and uncertainty in small-area estimates.

## Application to Swedish registry data

We illustrate the proposed framework using Swedish population-based registry data on prostate cancer.^9^ Data on tumor characteristics at diagnosis for incidence cases during 2018-2019 were obtained from the National Prostate Cancer Register, which captures more than 98% of all prostate cancer diagnoses in Sweden. Each case was categorized as late-stage (locally advanced or metastatic cancer at diagnosis) or early-stage (low-risk) following previously established definitions.

Sweden’s healthcare system is organized into 21 self-governing regions that manage prevention, screening, and diagnostic services.^37^ A hierarchical, small-area division into 5984 Demographic Statistical Areas (DeSO) was launched in 2018 to facilitate monitoring of segregation and socioeconomic conditions. The DeSOs vary in population size (between 650 and 4250 [year 2018]), urbanization, and socioeconomic composition, providing a relevant setting for small-area spatial modeling of stage-specific cancer incidence.

Individual-level information on age, income, and residential neighborhood was retrieved from Statistics Sweden’s population registries and linked to cancer outcomes through unique personal identification numbers. The corresponding population denominators for incidence calculations were derived from the same registers, enabling estimation of ASIRs by stage and small area. All data were aggregated to the DeSO-by-age-by-year strata before analysis. Men aged 50-84 years were included, consistent with the age range for which early detection through prostate-specific antigen testing is relevant.^21^ ASIRs were calculated using the national age distribution (Table S2). During 2018-2019, 4223 men were diagnosed with late-stage prostate cancer and 4891 with early-stage cancer. The corresponding national ASIRs were 117.4 and 136.0 per 100 000 person-years, respectively.

All bivariate models included an age-by-outcome interaction to allow stage-specific age effects, reflecting potential differences in age-specific incidence patterns between early- and late-stage disease. In contrast, the effect of calendar year was modeled as a common effect across outcomes because exploratory analyses indicated no substantial outcome-specific temporal variation, and a shared effect improved parsimony. Detailed model specifications and INLA implementation are provided in Table S1. Code and a synthetic dataset are available in the replication repository described in Appendix Section D. See Figures S1–S2 and Table S4 for synthetic data validation results.

Results from this Swedish prostate cancer application are summarized below. Model performance was first compared across bivariate specifications (Models 2-4) using DIC (Table 1). Models 3 and 4 substantially improved model fit compared with Model 2 ($\Delta \mathrm{DIC}\approx 160$), indicating that early- and late-stage incidence rates do not arise from a single underlying spatial process. Model 4 provided the best fit and was selected for subsequent analyses.

**Table 1:** Model performance and estimated cross-outcome correlations from alternative spatial formulations.

**Model**	**DIC**	**Cross-outcome correlation (** $\boldsymbol{\rho}$ **)** ^**a**^
Model 2: shared structured spatial effect	65 929.72	${\rho}_u=1$ by construction
Model 3: exchangeable correlated spatial fields	65 772.96	*ρ* : 0.227 (95% Crl: −0.016, 0.454)
Model 4: separate structured and unstructured components (BYM2)	65 768.84	${\rho}_u$ : 0.227 (95% Crl: −0.014, 0.451) ${\rho}_v$: 0.016 (95% Crl: −0.960, 0.955)

Abbreviations: BYM2, Besag–York–Mollié 2; Crl, credible interval; DIC, deviance information criterion.

a ${\rho}_u$ denotes the correlation between the structured spatial components; ${\rho}_v$ denotes the correlation between the unstructured components.

In Model 4, the estimated cross-outcome correlation for the structured spatial field was moderate (${\rho}_u\approx 0.227$; 95% credible interval: $\left[-0.014,0.451\right]$), indicating partial but not complete spatial overlap between early- and late-stage incidence patterns (Table 1). The correlation for the unstructured component in Model 4 was close to zero (${\rho}_v\approx 0.016$, 95% credible interval: $\left[-0.960,0.955\right]$), suggesting little residual similarity beyond the spatially structured field. Taken together, these results support Model 4 as the most appropriate specification for subsequent inference and priority-area mapping.

**Figure 2:**
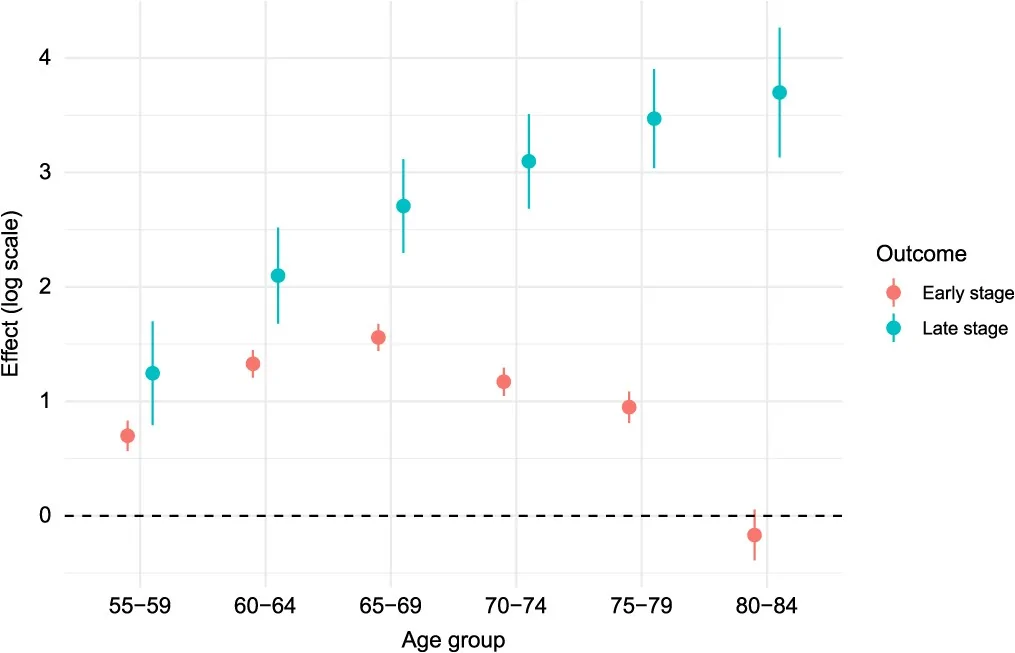
Posterior mean estimates and 95% credible intervals for age-group effects on early- and late-stage prostate cancer incidence, derived from the preferred bivariate BYM2 model (Model 4). Effects are expressed on the log scale relative to the reference group aged 50-54 years. Abbreviation: BYM2, Besag–York–Mollié 2.

A joint Wald test strongly rejected the null hypothesis of no age by outcome interaction (*P* < .001), confirming that the age gradient varies significantly across outcomes. Figure 2 presents the posterior mean estimates and 95% credible intervals for the age-group effects derived from Model 4 (log scale, reference = 50-54 years). Late-stage incidence increases steadily with age, reaching the highest levels among men aged 80-84 years, whereas early-stage incidence peaks around age 65-69 before declining at older ages.

**Figure 3:**
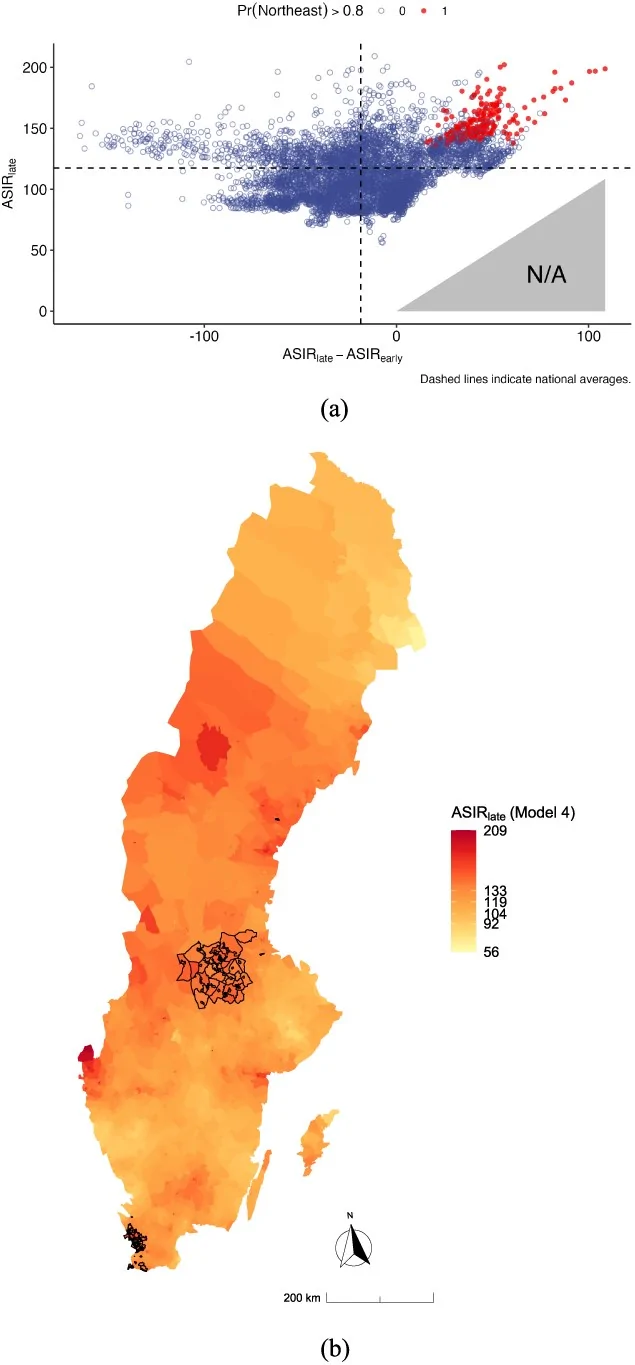
Identification of priority areas under the bivariate spatial model. (A) Joint posterior classification of DeSOs based on ${\mathrm{ASIR}}_{\mathrm{late}}$ and late-early ASIR difference (Model 4). Points in the upper-right quadrant with posterior probability larger than 0.8 are designated as priority areas. Dashed lines indicate national averages. (B) Geographic distribution of these priority DeSOs across Sweden. Shading represents the posterior median ${\mathrm{ASIR}}_{\mathrm{late}}$, with black borders highlighting priority areas. The map links the statistical criterion to its spatial visualization, revealing regional clusters of elevated late-stage incidence and diagnostic gaps. Abbreviations: ASIRs, age-standardized incidence rates and DeSO, Demographic Statistical Areas.

Posterior estimates from Model 4 were then used to identify areas with elevated late-stage incidence and insufficient early detection activity. Figure 3A shows the joint distribution of DeSO-level posterior estimates from the Model 4. As expected, most DeSOs cluster near the national average, while priority areas (red) concentrate in the upper-right quadrant, reflecting locations with both high late-stage incidence and large late-early differences (consistent with Figure 1). The corresponding map (Figure 3B) shows distinct clusters of disadvantaged DeSOs, most prominently in the southern and central Sweden, and 1 isolated area in the north.

**Figure 4:**
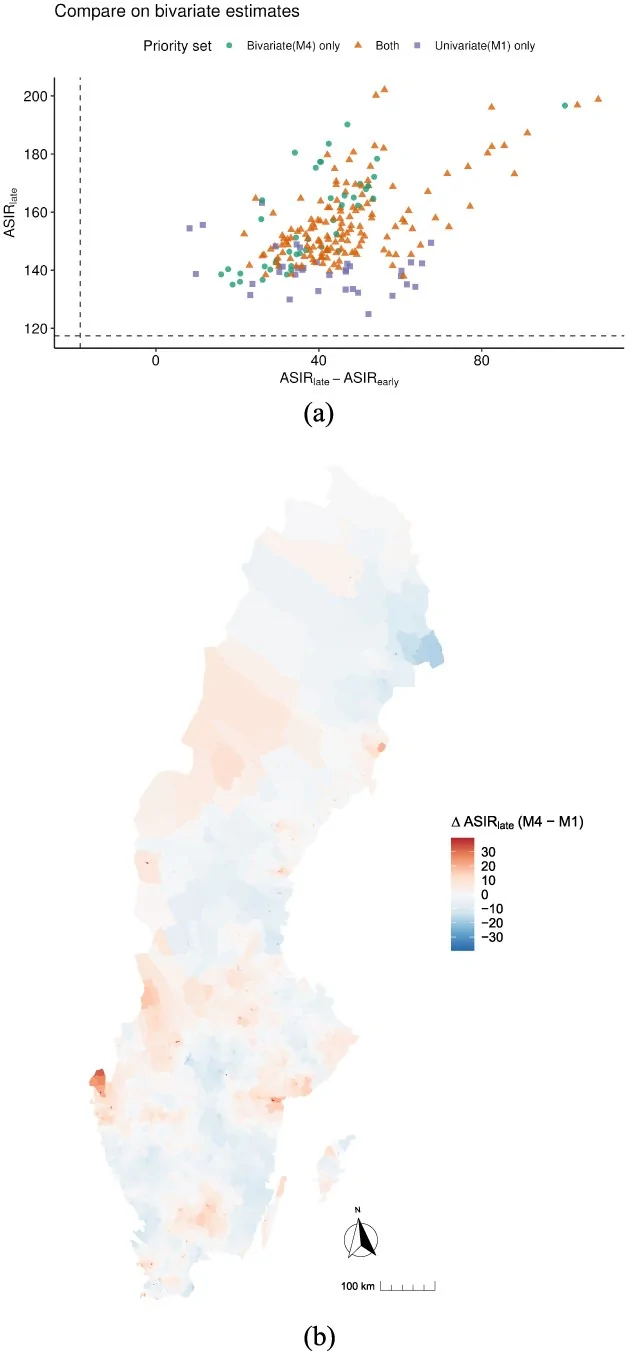
Comparison between univariate and bivariate spatial modeling results. (A) Comparison of DeSO-level classifications under the univariate (Model 1) and bivariate (Model 4) analyses. Different point symbols indicate areas identified as priority by both models, by Model 4 only, or by Model 1 only. The estimates are based on Model 4. Dashed lines represent national averages. (B) Difference in posterior median ${\mathrm{ASIR}}_{\mathrm{late}}$ between Model 4 and Model 1 ($\Delta ={\mathrm{ASIR}}_{\mathrm{late},\mathrm{Model}4}-{\mathrm{ASIR}}_{\mathrm{late},\mathrm{Model}1}$). Positive differences indicate locations where the bivariate model increased estimated incidence relative to the univariate model, whereas negative differences indicate locations where estimated incidence was reduced under joint bivariate modeling. The smoother, more coherent pattern under Model 4 reflects the expected effect of spatial and cross-outcome smoothing. Abbreviations: ASIRs, age-standardized incidence rates and DeSO, Demographic Statistical Areas.

We then compared the bivariate (Model 4) and univariate (Model 1) classifications to evaluate the influence of joint modeling on area identification. As shown in Figure 4A, 145 DeSOs were consistently identified by both models, 44 only under the bivariate specification, and 35 only under the univariate model. The corresponding difference map (Figure 4B) visualizes the change in posterior median late-stage ASIRs between models. Overall, Model 4 yielded a more coherent and epidemiologically plausible spatial pattern, reducing isolated extremes and strengthening inference for clusters supported by both outcomes.

**Figure 5:**
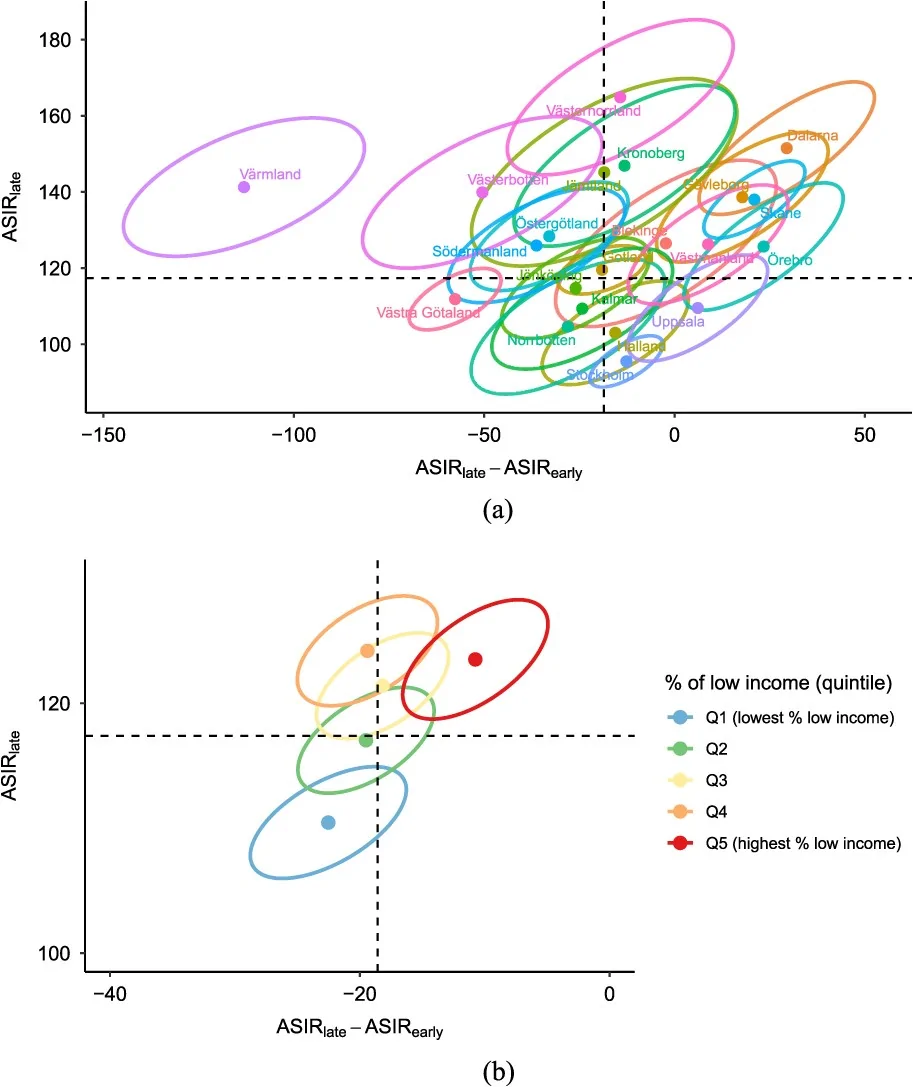
Regional and socioeconomic patterns in stage-specific incidence. (A) Joint posterior distribution of DeSO-level ASIRs for late-stage cancer and the difference between late- and early-stage ASIRs, stratified by regions. Points represent posterior medians across 1000 draws, and ellipses denote 80% joint credible regions derived from the empirical posterior covariance (see Appendix Section E for details). Dashed lines indicate observed national averages. (B) Joint posterior distribution of DeSO-level ASIRs for late-stage cancer and the difference between late- and early-stage ASIRs, stratified by quintiles of the proportion of low-income residents (Q1 = least deprived, Q5 = most deprived). Points represent posterior medians across 1000 draws, and ellipses denote 80% joint credible regions derived from the empirical posterior covariance (see Appendix for details). Dashed lines indicate observed national averages. Abbreviations: ASIRs, age-standardized incidence rates and DeSO, Demographic Statistical Areas.

Figure 5A presents the regional-level comparison of late-stage incidence and the late-early difference. Värmland appears distinct in the upper-left quadrant, characterized by moderately high late-stage incidence but a very small late-early gap, reflecting high early-stage incidence. A likely explanation is that, in 2015, region Värmland began actively informing men aged 50-70 about prostate-cancer testing, preceding similar organized efforts elsewhere in Sweden.^38^ Because Sweden’s 21 regions independently manage preventive and diagnostic services, this example illustrates how regional initiatives may elevate diagnostic activity. Figure 5B presents the corresponding pattern by socioeconomic-status quintiles, illustrating that areas with lower socioeconomic status tend to have both higher late-stage incidence and less favorable stage distribution, consistent with previously observed disparities. An interactive map displaying all model-specific classifications is also provided in the Supplementary material.

Lastly, we compared our proposed framework with an alternative conceptualization based on total incidence and the proportion of late-stage diagnoses,^13^ which identified substantially fewer priority areas (0-13 vs. 180-189). A similar pattern was observed under a simplified sequential estimation approach (total incidence modeled as a Poisson model and the late-stage proportion using a binomial model). Full results are provided in Appendix Section C and Table S3.

## Discussion

This study introduces a Bayesian bivariate spatial modeling framework for jointly estimating small-area incidence of early- and late-stage cancers. By allowing correlation both across space and between outcomes, the approach improves precision of local estimates while preserving interpretable epidemiologic structure. Compared with conventional univariate models, the bivariate BYM2 formulation borrows strength not only from spatial neighbors but also from related outcomes, yielding smoother and more stable posterior surfaces in data-sparse areas. The framework can be generalized to other diseases characterized by correlated outcomes, multiple severity levels, or competing diagnostic pathways, and to settings beyond Sweden, particularly in countries with large geographic or socioeconomic variation in healthcare delivery.

The latent spatial components estimated in our models represent unmeasured influences that vary across areas, reflecting a combination of contextual factors (eg, environmental exposures, healthcare organization, socioeconomic conditions) and individual-level characteristics that may cluster geographically when residential segregation is present. For example, men of African descent show higher risk for prostate cancer and may also reside disproportionately in socioeconomically disadvantaged neighborhoods with limited access to diagnostic services,^39^ leading to overlapping contextual and compositional effects on observed incidence patterns.

The structured spatial component captures influences that vary smoothly across space, while the unstructured component reflects more localized or discontinuous variation that may arise.^40^^,^^41^ Importantly, the distinction between “structured” and “unstructured” is not absolute but depends on the level of spatial analysis and the scale at which the underlying mechanisms operate.^42^^,^^43^ A process that appears spatially continuous at a fine scale may appear unstructured when aggregated to broader administrative units. By estimating both components jointly, the bivariate BYM2 formulation flexibly captures spatial dependence where present while allowing for residual heterogeneity.

Conceptualizing the models in this way clarifies their hierarchy: Model 2 assumes a single spatial process, Model 3 allows correlated but distinct processes, and Model 4 provides the most flexible specification by separating structured and unstructured components. This hierarchy acknowledges that early- and late-stage incidence may be shaped by overlapping but not identical mechanisms. In our application, the moderate positive structured correlation (${\rho}_u\approx 0.23$) suggests partial overlap in spatial determinants of early- and late-stage incidence, likely reflecting a combination of shared contextual factors and differences in diagnostic activity.

Mapping both late-stage incidence alongside the late-minus-early difference provides a practical way to pinpoint areas where enhanced early detection may yield the greatest benefits. The bivariate approach refines this inference by integrating information across outcomes, leading to more coherent identification of priority areas. In our application to prostate cancer, Model 4 revealed additional disadvantaged areas embedded within broader high-risk clusters that a univariate analysis might overlook. Such evidence can inform regional cancer-control programs seeking to allocate diagnostic resources equitably.

Socioeconomic contextualization further strengthens interpretation. The overlap between priority DeSOs and areas of lower socioeconomic conditions suggests that structural and social barriers may contribute to delayed detection. These findings are consistent with previous reports of geographic and socioeconomic inequalities in screening uptake and diagnostic yield across Sweden and other high-income countries.^4^^,^^9^^,^^44^^,^^45^ Integrating bivariate spatial modeling with socioeconomic data can thus help identify not only where diagnostic activity is insufficient but also how social gradients and contextual factors are reflected in spatial disparities.

Our work builds upon multivariate Bayesian disease-mapping models developed to jointly model correlated outcomes and borrow strength across related spatial processes.^14-19^ Conceptually, these frameworks correspond most closely to our Model 3, which assumes a shared or exchangeable spatial surface. To our knowledge, however, this study presents the first applied implementation of a bivariate BYM2 parameterization that allows each outcome its own structured and unstructured spatial components with explicit cross-outcome correlation parameters. This formulation, represented by our Model 4, provides a more flexible and interpretable way to distinguish spatially structured and heterogeneous, local sources of association between outcomes, integrating epidemiologic reasoning about diagnostic activity and disease burden into a coherent Bayesian framework. More importantly, we apply this framework to a new epidemiologic context, focusing on stage-specific cancer incidence to quantify geographic inequalities in diagnostic activity. Further research could extend this bivariate spatial framework to spatiotemporal settings^19^^,^^46^ to examine how diagnostic behaviors evolve over time and extend it to multiple cancer types and additional severity categories. The 0.8 posterior-probability threshold offers a transparent decision rule. Yet, alternative cut-offs may be explored depending on public-health priorities.

Finally, our findings highlight that differences between alternative approaches^13^ arise not only from model specification but also from how estimated quantities are conceptualized to represent disease burden and diagnostic activity. In our application, we observed broadly similar spatial patterns across formulations but substantial differences in threshold-based identification of priority areas, primarily driven by differences in conceptualization when translating model outputs into public health decisions.

In summary, the proposed bivariate BYM2 framework provides a generalizable approach for quantifying geographic inequalities in cancer detection. By linking statistical precision with epidemiologic interpretation, it supports evidence-based strategies to improve equity in early diagnosis.

## Ethics declaration

The register-based study (ie, the empirical example) was approved by the Swedish Ethical Review Authority (reference number: 2023-07767-01).

## Supplementary Material

Web_Material_kwag106

## Data Availability

The empirical data used for demonstration was extracted from the Prostate Cancer data Base Sweden (PCBaSe), which is based on the National Prostate Cancer Register (NPCR) of Sweden and linkage to several health-data registers and Statistics Sweden’s population registers. This study was approved by the Swedish Ethical Review Authority (reference number: 2023-07767-01). Researchers interested in accessing the original register-based data may apply through the Swedish Ethical Review Authority and PcBaSe, in accordance with the national regulations. Replication materials, including R code and a synthetic dataset enabling reproduction of the analysis workflow, are publicly available via the Open Science Framework (OSF) at https://osf.io/3gjzt/.
