# Supplementary material for: A Bayesian bivariate spatial modeling framework for stage-specific cancer incidence and targeting of screening efforts
Source: Am J Epidemiol. 2026 May 19;195(9):2430–9. doi: 10.1093/aje/kwag106 (PMC13537854; doi:10.1093/aje/kwag106)
Supplement: Supplementary file 1 [file web_material_kwag106.zip › appendix_final.docx]

**Supplementary Appendix for:**

***A Bayesian Bivariate Spatial Modeling Framework for Stage-Specific Cancer Incidence and Targeting of Screening Efforts***

Qinyun Lin, Carl Bonander, Ulf Strömberg

**Section A:** Bivariate model specifications and INLA implementation, including:

- Table S1: Bivariate model specifications and INLA implementation

**Section B:** Age-standardization of incidence rates (post-estimation), including:

- Table S2: National age-standardization weights used to calculate ASIRs

**Section C:** Empirical comparison of estimation and conceptualization strategies, including

- Table S3: Comparison of priority areas identified under different estimation strategies (Dimension 1) and conceptualizations (Dimension 2)

**Section D:** Synthetic data generation and validation, including:

- Figure S1: Scatterplot comparing DeSO-level late-stage ASIR estimates derived from the observed and synthetic datasets.
- Figure S2: Priority classification under the synthetic dataset.
- Table S4: Model comparison statistics for the synthetic dataset.

**Section E:** Technical details for computation of joint posterior ellipses in Figure 5 (both empirically and analytically)

**Section A. Bivariate model specifications and INLA implementation**

The four spatial models considered in the analysis are summarized in Table S1. The table describes the spatial structure, cross-outcome correlation assumptions, fixed effects, corresponding INLA implementation, and prior specifications used in each model.

**Table S1.** Bivariate model specifications and INLA implementation

| **Model** | **Spatial structure** | **Cross-outcome correlation** | **Fixed effects (all models)** | **INLA terms** | **Key priors** |
| --- | --- | --- | --- | --- | --- |
| **M1:** *two univariate models* | BYM2 per outcome (structured + iid) | **None** (separate fits) | Intercept, **age_cat**, **Year**, (no outcome term here because split by outcome) | f(spatial_struct, model="bym2", graph=H, scale.model=TRUE) | prec ~ pc.prec(1,0.01), phi ~ pc(0.5,0.5) |
| **M2:** *Shared structured + outcome-specific iid* | **Shared** structured (ICAR); **iid per outcome** | **None** (iid independent across outcomes) | -1 + outcome_f + age_cat + Year + outcome_f:age_cat | f(spatial_struct, model="besag", graph=H, scale.model=TRUE, constr=TRUE) + f(spatial_unstr, model="iid", replicate=group) | prec ~ pc.prec(1,0.01) for both terms |
| **M3:** *Exchangeable bivariate BYM2* | **One BYM2 field duplicated across outcomes** (structured+iid together) | **Single** ρ (exchangeable) for the combined BYM2 field |  | f(spatial_struct, model="bym2", graph=H, scale.model=TRUE, group=group, control.group=list(model="exchangeable")) | prec ~ pc.prec(1,0.01), phi ~ pc(0.5,0.5) |
| **M4:** *Full bivariate BYM2-type specification (two correlations)* | **Besag (structured) per outcome** + **iid per outcome** | **Two** ρ’s: ($\rho_{u}$) (structured), ($\rho_{v}$) (iid) |  | f(spatial_struct, model="besag", graph=H, scale.model=TRUE, constr=TRUE, group=group, control.group=list(model="exchangeable")) + f(spatial_unstr, model="iid", group=group, control.group=list(model="exchangeable")) | prec ~ pc.prec(1,0.01) for both terms |

*Note*. We used penalized-complexity (PC) priors for all random-effect precision parameters (τ). These priors are parameterized in terms of the standard deviation $\sigma= 1/\sqrt{\tau}$. Specifically, we set $P\left( \sigma>1 \right)=0.01$ (i.e., pc.prec(param = c(1, 0.01)), which weakly shrinks extreme spatial variability while allowing substantial deviation when supported by the data. All models used identical PC priors for precision parameters. For the BYM2 specifications (Models 1 and 3), a PC prior was additionally assigned to the mixing parameter $\phi$. The exchangeable correlation parameters in Models 3 and 4 were estimated using the default R-INLA prior associated with control.group = list(model = "exchangeable"), "), which is a weakly informative normal prior on INLA’s internal correlation scale (mean 0, precision 0.2), transformed internally to the correlation parameter.

**Section B. Age-standardization of incidence rates (post-estimation)**

Age standardization was applied after model estimation. Age-specific incidence rates predicted from the fitted model were weighted according to the national age distribution of the study population (ages 50–84 years), corresponding to direct standardization. This yields model-based age-standardized incidence rates (ASIRs) for both early- and late-stage cancer.

In practice, for each DeSO × age group × calendar year stratum, the fitted model provides posterior estimates of age-specific incidence rates for early- and late-stage outcomes. ASIRs were calculated by weighting the model-predicted age-specific incidence rates (posterior medians) using the national age distribution. This produces absolute, age-adjusted stage-specific incidence rates and their difference, which serve as comparable population-level summary measures for identifying priority areas.

**Table S2.** National age-standardization weights used to calculate ASIRs.

| **Age group (years)** | **Weight** |
| --- | --- |
| 50–54 | 0.193 |
| 55–59 | 0.171 |
| 60–64 | 0.158 |
| 65–69 | 0.150 |
| 70–74 | 0.153 |
| 75–79 | 0.110 |
| 80–84 | 0.066 |

***Note*.** Weights were derived from the national male population distribution for ages 50–84 years during the study period.

**Section C. Empirical comparison of estimation and conceptualization strategies**

We compared our proposed framework with an alternative formulation based on total incidence and the proportion of late-stage diagnoses, as considered in previous work on stage-specific cancer incidence. To clarify the sources of differences across approaches, we distinguish between two conceptually separate dimensions:

- Dimension 1: Estimation, i.e., how stage-specific incidence rates are modeled and estimated; and
- Dimension 2: Conceptualization, i.e., how these estimates are combined to define disease burden, stage distribution, and identify priority areas.

These dimensions are conceptually distinct. For any given estimation strategy, different conceptualizations can be applied to the same underlying estimates.

To illustrate this distinction, we compared two conceptualizations:

(i) a framework based on late-stage incidence and the late–early difference (our proposed approach for prostate cancer), and

(ii) an alternative formulation based on total incidence and the proportion of late-stage diagnoses,

under two estimation strategies:

(A) our primary modeling approach (univariate and bivariate Poisson spatial models for early- and late-stage incidence), and

(B) a sequential approach (Poisson model for total incidence and binomial model for the proportion of late-stage diagnoses).

The results are summarized in Table S3.

**Table S3**. Comparison of priority areas identified under different estimation strategies (Dimension 1) and conceptualizations (Dimension 2)

|  | **Dimension 2: Conceptualization for identifying priority areas** | |
| --- | --- | --- |
| **Dimension 1: Estimation** | Late-stage + (late – early) | Total + proportion late (alternative formulation) |
| **(A) Our models** (two Poisson spatial models; univariate or bivariate) | 180 (uni, M1); 189 (biv, M4) | 0 (uni, M1); 13 (biv, M4) |
| **(B) Sequential approach** (simplified formulation) | 165 | 2 |

*Note*. This table summarizes the number of priority areas identified using a posterior probability cutoff of 0.8 under different combinations of estimation strategies (Dimension 1) and conceptualizations (Dimension 2). Dimension 1 refers to how stage-specific incidence rates are estimated, while Dimension 2 refers to how these estimates are combined to define priority areas.

Across estimation strategies, the choice of conceptualization (Dimension 2) had a substantial impact on the number of identified priority areas. The late-stage + difference framework identified a considerable number of areas (165–189 areas), whereas the total incidence + proportion formulation identified very few areas (0–13 areas).

On the other hand, differences between estimation strategies (Dimension 1) were comparatively modest, suggesting that the conceptualization step plays a dominant role in determining threshold-based classification of priority areas.

Despite these differences in classification, we observed a high degree of concordance in the ranking of areas. In particular, posterior probabilities derived from the sequential formulation and our proposed framework were strongly correlated (Spearman correlation ≈ 0.86). This indicates that both approaches capture broadly similar underlying spatial patterns, but differ in how these patterns are translated into binary decisions.

The discrepancy is primarily driven by the use of proportions in the alternative formulation. The proportion of late-stage diagnoses is a non-linear transformation of the underlying rates, which compresses variation toward intermediate values. As a result, posterior probabilities are less likely to exceed predefined thresholds simultaneously, even when relative differences between areas are preserved. This effect is particularly pronounced when identifying areas exceeding two thresholds jointly.

The proposed framework, on the other hand, is based on absolute incidence measures, which more directly reflect population-level disease burden and are less sensitive to such compression effects. Taken together, these results highlight that the choice of conceptualization (Dimension 2) has a greater influence on priority area identification than the choice of estimation strategy (Dimension 1), even when underlying spatial patterns are similar.

**Section D. Synthetic data generation and validation**

To facilitate reproducibility, we generated a synthetic dataset based on the fitted spatial model. Specifically, we first fitted the most flexible model (Model 4) to the observed data. We then drew a single posterior sample of the model parameters and latent effects and generated synthetic case counts conditional on the sampled latent structure. This approach preserves the joint spatial dependence structure captured by the fitted model while ensuring that the shared dataset does not contain identifiable registry information.

Replication materials, including the synthetic dataset and R scripts used in the analyses, are publicly available in an OSF repository at <https://osf.io/3gjzt/>. All analyses included in the reproducibility repository were conducted using this synthetic dataset to demonstrate the analytical workflow.

To assess the similarity between the observed and synthetic datasets, we compared several summary properties, including the distribution of age-standardized incidence rates (ASIRs), the correlation of DeSO-level ASIRs between datasets, and the overlap of priority areas identified using the proposed classification rule.

Figure S1 compares DeSO-level ASIR estimates between the observed and synthetic datasets (using late-stage ASIR as illustration). The Spearman correlation between observed and synthetic DeSO-level ASIRs was approximately 0.78 for both late-stage incidence and the late–early incidence difference, suggesting that the synthetic dataset preserves much of the spatial heterogeneity observed in the original data.

We also examined whether priority areas identified using the synthetic dataset were similar to those identified from the observed data. Among the top 1000 DeSOs ranked by late-stage ASIR, 565 overlapped between the observed and synthetic datasets. Among the top 200 DeSOs ranked by late-stage ASIR, 62 overlapped between the two datasets.

Applying the same classification rule used in the main analysis (posterior probability threshold of 0.8), the synthetic data identified 229 priority areas compared with 189 in the observed dataset. Although the exact set of areas differs somewhat, the overall spatial patterns and concentration of high-incidence areas are similar. Figure S2 illustrates the joint classification of DeSOs based on late-stage ASIR and the late–early ASIR difference under the synthetic dataset.

Finally, we confirmed that the relative performance of the competing spatial models remained consistent when applied to the synthetic dataset. Model comparison metrics showed that the full bivariate model (M4) provided the best fit, with lower DIC values than Models M2 and M3 (Table S4). This mirrors the conclusions obtained using the observed data.

**Figure S1**. Scatterplot comparing DeSO-level late-stage ASIR estimates derived from the observed and synthetic datasets. The red line denotes the 45-degree line indicating perfect agreement between the two datasets.

**Figure S2**. Priority classification under the synthetic dataset.

Scatterplot of DeSO-level late-stage ASIR and the late–early ASIR difference. Dashed lines indicate the classification thresholds used in the analysis. Red points denote areas classified as priority areas using the posterior probability criterion $Pr\left( \text{Northeast} \right)>0.8$.

**Table S4**. Model comparison statistics for the synthetic dataset.

| **Model** | **DIC** |
| --- | --- |
| Model 2: Shared structured spatial effect | 64946.64 |
| Model 3: Exchangeable correlated spatial fields | 64775.92 |
| Model 4: Separate structured and unstructured components (BYM2) | 64762.26 |

**Abbreviations**: DIC, deviance information criterion.

**Section E. Technical appendix**

**Computation of Joint Posterior Ellipses (Figure 5)**

For each posterior draw ($m=1,\ldots,1000)$, we computed median DeSO-level age-standardized incidence rates (ASIRs) for early- and late-stage cancers, aggregated by both region ($n = 21$) and income quintile ($q=1, \ldots,5$). Within each grouping, we then calculated the empirical covariance matrix between ${ASIR}_{late}$ and ${ASIR}_{late}-{ASIR}_{early}$ across the 1,000 posterior samples. Ellipses in Figure 5 represent the 80% joint credible regions derived from these covariance matrices, centered on the posterior median values.

While the ellipses in Figure 5 were derived empirically from posterior draws, the joint covariance matrix can also be computed analytically, as illustrated below.

Suppose we have $K$ age strata, each with $N_{j}$ individuals (or person-years) for $j=1,\ldots,K$. In each stratum $j$, we observe counts $(a_{\mathcal{l,}j},a_{\mathcal{e,}j},a_{\mathcal{o,}j}$) for three mutually exclusive categories: late-stage cancer at diagnosis, $\mathcal{l}$, early-stage cases, $\mathcal{e}$, and the remainder of the population $\mathcal{o}$ (i.e., none and, possibly, other/unknown), such that $a_{\mathcal{l,}j}+a_{\mathcal{e,}j}+a_{\mathcal{o,}j}=N_{j}$.

We assume these counts follow a multinomial distribution with parameters ($N_{j};p_{\mathcal{l,}j},p_{\mathcal{e,}j},p_{\mathcal{o,}j})$. Define the estimated age-specific rates $\hat{r}_{\mathcal{l,}j}={a_{\mathcal{l,}i}}/{N_{j}}$ and $\hat{r}_{e,j}={a_{\mathcal{e,}j}}/{N_{j}}$. Let $w_{j}>0$ be a standard weight for stratum $j$, with $\sum_{j=1}^{K} w_{j}=1$. The age-standardized incidence rate for late-stage cancer is given by ${ASIR}_{\mathcal{l}}=\sum_{j=1}^{K} w_{j}\hat{r}_{\mathcal{l,}j}$, and similarly for early-stage cases, ${ASIR}_{\mathcal{e}}=\sum_{j=1}^{K} w_{j}\hat{r}_{\mathcal{e,}j}$.

First, consider the variance of ${ASIR}_{\mathcal{l}}$. Assuming the counts in different strata are independent, the variance of the sum is simply the sum of the stratum-specific variances. Hence,

$$\mathrm{Var}\left( {ASIR}_{\mathcal{l}} \right)=\mathrm{Var}\left( \sum_{j=1}^{K} w_{j}\hat{r}_{\mathcal{l,}j} \right)$$

$$=\sum_{j=1}^{K} w_{j}^{2}\mathrm{Var}\left( \frac{a_{\mathcal{l,}j}}{N_{j}} \right)$$

$$=\sum_{j=1}^{K} w_{j}^{2}\frac{\mathrm{Var}\left( a_{\mathcal{l,}j} \right)}{N_{j}^{2}}.$$

(1)

Within the $j$th stratum, $a_{\mathcal{l,}j} \sim\mathrm{Multinomial}(N_{j},p_{\mathcal{l,}j}$), which gives $\mathrm{Var}\left( a_{\mathcal{l,}j} \right)=N_{j}p_{\mathcal{l,}j}\left( 1-p_{\mathcal{l,}j} \right).$ Plugging into Equation (1) gives

$$\mathrm{Var}\left( {ASIR}_{\mathcal{l}} \right)=\sum_{j=1}^{K} w_{j}^{2}\frac{N_{j}p_{\mathcal{l,}j}\left( 1-p_{\mathcal{l,}j} \right)}{N_{j}^{2}}$$

$$=\sum_{j=1}^{K} w_{j}^{2}\frac{p_{\mathcal{l,}j}\left( 1-p_{\mathcal{l,}j} \right)}{N_{j}},$$

(2)

where $p_{\mathcal{l,}j}$ is replaced by $\hat{r}_{\mathcal{l,}j}$ in practice to obtain a plug-in estimator. Note that for rare diseases, $p_{\mathcal{l,}j}\left( 1-p_{\mathcal{l,}j} \right)\approx p_{\mathcal{l,}j}$, which coincides with the Poisson-based variance $N_{j}p_{\mathcal{l,}j}$ for the count $a_{\mathcal{l,}j}$.

Next, consider ${ASIR}_{\mathcal{l}}-{ASIR}_{e}$. By linearity,

$$\mathrm{Var}\left( {ASIR}_{\mathcal{l}}-{ASIR}_{e} \right)=\sum_{i=j}^{K} w_{j}^{2}\mathrm{Var}\left( \frac{a_{\mathcal{l,}j}}{N_{j}}-\frac{a_{e,j}}{N_{j}} \right)=\sum_{j=1}^{K} w_{j}^{2}\frac{\mathrm{Var}\left( a_{\mathcal{l,}j} \right)+\mathrm{Var}\left( a_{e,j} \right)-2\mathrm{Cov}\left( a_{\mathcal{l,}j},a_{e,j} \right)}{N_{j}^{2}}$$

(3)

Because the random vector $(a_{\mathcal{l,}j},a_{\mathcal{e,}j},a_{\mathcal{o,}j}$) is multinomial, the covariance is always negative and has a closed-form solution where $\mathrm{Cov}\left( a_{\mathcal{l,}j},a_{e,j} \right)=-N_{j}p_{\mathcal{l,}j}p_{e,j}.$ Plugging variance and covariance expressions into Equation (3) gives:

$$\mathrm{Var}\left( {ASIR}_{\mathcal{l}}-{ASIR}_{e} \right)=\sum_{j=1}^{K} w_{j}^{2}\frac{N_{j}p_{\mathcal{l,}j}\left( 1-p_{\mathcal{l,}j} \right)+N_{j}p_{e,j}\left( 1-p_{e,j} \right)-2\left( -N_{j}p_{\mathcal{l,}j}p_{e,j} \right)}{N_{j}^{2}}$$

$$=\sum_{j=1}^{K} w_{j}^{2}\frac{N_{j}\left( p_{\mathcal{l,}j}\left( 1-p_{\mathcal{l,}j} \right)+p_{e,j}\left( 1-p_{e,j} \right)+2p_{\mathcal{l,}j}p_{e,j} \right)}{N_{j}^{2}}$$

$$=\sum_{j=1}^{K} w_{j}^{2}\frac{p_{\mathcal{l,}j}\left( 1-p_{\mathcal{l,}j} \right)+p_{e,j}\left( 1-p_{e,j} \right)+2p_{\mathcal{l,}j}p_{e,j}}{N_{j}},$$

(4)

where, as above, $p_{\mathcal{l,}j}$ and $p_{e,j}$ are replaced by $\hat{r}_{\mathcal{l,}j}$ and $\hat{r}_{\mathcal{e,}j}$ to obtain a plug-in estimator.

Finally, to construct a confidence ellipse for the bivariate vector (${ASIR}_{\mathcal{l}}-{ASIR}_{e},{ASIR}_{\mathcal{l}}$), we need to estimate the covariance matrix

$$\boldsymbol{\Sigma}\boldsymbol{=}\left( \begin{matrix} \mathrm{Var}\left( {ASIR}_{\mathcal{l}}-{ASIR}_{e} \right) & \mathrm{Cov}\left( {ASIR}_{\mathcal{l}}-{ASIR}_{e},{ASIR}_{\mathcal{l}} \right) \\ \mathrm{Cov}\left( {ASIR}_{\mathcal{l}}-{ASIR}_{e},{ASIR}_{\mathcal{l}} \right) & \mathrm{Var}\left( {ASIR}_{\mathcal{l}} \right) \end{matrix} \right)\boldsymbol{.}$$

The diagonal elements, $\mathrm{Var}\left( {ASIR}_{\mathcal{l}}-{ASIR}_{e} \right)$ and $\mathrm{Var}\left( {ASIR}_{\mathcal{l}} \right)$, can be estimated using the plug-in estimators of Equations (2) and (4), respectively. By linearity, the asymptotic expression for the off-diagonal covariance elements can also be written as

$$\mathrm{Cov}\left( {ASIR}_{\mathcal{l}}-{ASIR}_{e},{ASIR}_{\mathcal{l}} \right)=\mathrm{Cov}\left( {ASIR}_{\mathcal{l}},{ASIR}_{\mathcal{l}} \right)-Cov\left( {ASIR}_{\mathcal{l}},{ASIR}_{e} \right)$$

$$=\mathrm{Var}\left( {ASIR}_{\mathcal{l}} \right)-Cov\left( {ASIR}_{\mathcal{l}},{ASIR}_{e} \right)$$

$$=\sum_{j=1}^{K} w_{j}^{2}\frac{N_{j}p_{\mathcal{l,}j}\left( 1-p_{\mathcal{l,}j} \right)}{N_{j}^{2}}-\sum_{j=1}^{K} w_{j}^{2}\frac{-N_{j}p_{\mathcal{l,}j}p_{e,j}}{N_{j}^{2}}$$

$$=\sum_{j=1}^{K} w_{j}^{2}\frac{p_{\mathcal{l,}j}\left( 1-p_{\mathcal{l,}j} \right)}{N_{j}}-\sum_{j=1}^{K} w_{j}^{2}\frac{-p_{\mathcal{l,}j}p_{e,j}}{N_{j}}$$

$$=\sum_{j=1}^{K} w_{j}^{2}\frac{p_{\mathcal{l,}j}\left( 1-p_{\mathcal{l,}j} \right)+p_{\mathcal{l,}j}p_{e,j}}{N_{j}}$$

$$=\sum_{j=1}^{K} w_{j}^{2}\frac{p_{\mathcal{l,}j}\left( {1-p_{\mathcal{l,}j}+p}_{e,j} \right)}{N_{j}},$$

(5)

where second equality follows from the fact that $\mathrm{Cov}\left( {ASIR}_{\mathcal{l}},{ASIR}_{\mathcal{l}} \right)=\mathrm{Var}\left( {ASIR}_{\mathcal{l}} \right)$, the third by plugging in the closed-form expressions derived above; the rest follow by algebraic manipulation. Again, a simple plug-in estimator can be used: Under large‐sample conditions, (${{ASIR}_{\mathcal{l}}-{ASIR}_{e},ASIR}_{\mathcal{l}}$) is approximately bivariate normal with mean given by the sample estimates and covariance matrix $\hat{\boldsymbol{\Sigma}}$ given by plugging in $\hat{r}_{\mathcal{l,}j}$ and $\hat{r}_{\mathcal{e,}j}$.
